# The association of mean telomere length with all-cause, cerebrovascular and cardiovascular mortality

**DOI:** 10.1042/BSR20192306

**Published:** 2019-10-25

**Authors:** Yu-qing Huang, Kenneth Lo, Ying-qing Feng, Bin Zhang

**Affiliations:** Department of Cardiology, Guangdong Cardiovascular Institute, Hypertension Research Laboratory, Guangdong Provincial People’s Hospital, Guangdong Provincial Key Laboratory of Coronary Heart Disease Prevention, Guangdong Academy of Medical Sciences, South China University of Technology School of Medicine, Guangzhou 510080, China

**Keywords:** all-cause mortality, cardiovascular mortality, cerebrovascular mortality, Mean telomere length

## Abstract

Mean telomere length (MLT) is a marker of cell aging and may associate with age-related diseases. However, the relationship between MLT and mortality risk remains unclear. We aimed to investigate the relationship between MLT and all-cause, cerebrovascular and cardiovascular mortality among adults in United States. We analyzed data were from National Health and Nutrition Examination Survey (NHANES, 1999–2002) with follow-up data through 31 December 2015. Based on MLT, participants were categorized into low, middle and high groups. Multivariate Cox proportional hazards regression, subgroup analysis and generalized additive model (GAM) were performed by using hazard ratios (HRs) and 95% confidence intervals (CIs). A total of 7827 participants were included in analysis (48.18% male). After 158.26 months of follow-up on average, there were 1876 (23.97%), 87 (1.11%) and 243 (3.10%) onset of all-cause, cerebrovascular and cardiovascular mortality. After adjustment for potential confounders, using the low group as the reference, HRs for all-cause (0.87 and 0.86), cerebrovascular (0.75 and 0.75) and cardiovascular mortality (1.01 and 0.69) for the middle to high groups were not statistically significant (all *P*>0.05 for trend). MLT was non-linearly related to all-cause mortality but not to cerebrovascular and cardiovascular mortality. It was the first study to demonstrate the non-linear relationship between MLT and all-cause mortality.

## Introduction

With the development of life sciences and technology, a large number of experiments indicated that circulating biomarkers such as telomere length might associate with the risk of mortality [1]. Specifically, cardiovascular mortality has been an increasing burden for public health, more efforts are needed to elucidate the associated risk factors.

Telomeres are the caps and repetitive nucleotide sequences located at the end of linear chromosomes of most eukaryotic organisms [2]. Telomere is also a necessary and vital part of human cells which shortens throughout the lifespan that affect our cells age in humans [3]. Telomere length has been acting as an important biomarker of biological age and was linked to the etiology of age-related diseases, such as atherosclerosis [4], Alzheimer’s disease [5], stroke [6] and coronary heart disease [7]. Short leukocyte telomere length is a hallmark characteristic of aging and might predict mortality independent of chronical age [8,9]. Though several epidemiological and clinical studies showed a relationship between shorter telomere length and the risk of morbidity and mortality, the results remained elusive and heterogeneous [10–16]. In addition, to the best of our knowledge, most of the previous studies were conducted among elderly or with small sample size. Evidence from a large sample of a well-designed cohort study among general population is lacking. Therefore, we aim to investigate the potential association between mean telomere length (MTL) and all-cause, cerebrovascular and cardiovascular mortality using the data from the National Health and Nutrition Examination Survey (NHANES).

## Materials and methods

### Study design and study population

**Figure 1:**
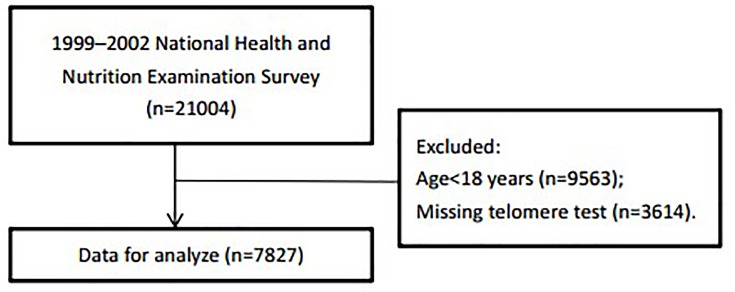
The research flow chart

The NHANES is a national representative survey of the civilian, non-institutionalized U.S. population conducted by the Centers for Disease Control and Prevention (CDC) [15]. In the present study, we included 7827 participants aged ≥ 18 and had the telomere test results from NHANES (1999–2002) (Figure 1). The survey protocol was approved by the Institutional Review Board of the CDC. All participants provided written informed consent.

### Measurements

Questionnaires were distributed to participants to acquire demographics information, including smoking, drinking, previous history of diseases, medication history. Physical assessments and laboratory tests were performed to examine systolic blood pressure (SBP), diastolic blood pressure (DBP), height, weight, fasting blood glucose (FBG), total cholesterol (TC), triglyceride (TG), low-density lipoprotein cholesterol (LDL-C), high-density lipoprotein cholesterol (HDL-C), C-reactive protein (CRP). Body mass index (BMI) was defined as mass (kg) divided by the square of height (m^2^). Estimated glomerular filtration rate (ml/min/1.73 m^2^) (eGFR) was calculated through the formula: 186 × (SCr)^−1.154^ × (age)^−0.203^ × [0.724 if female] × [1.212 if Black], where SCr is serum creatinine (mg/dl) [17]. An SCr level in μmol/l was converted into mg/dl by dividing them by 88.4. The telomere length assay was performed by using the quantitative polymerase chain reaction (qPCR) method to measure telomere length relative to standard reference DNA (T/S ratio), as described in detailed previously [18]. Detailed procedures on questionnaires and testing methods can be found on the website (https://wwwn.cdc.gov/nchs/nhanes/Default.aspx and https://wwwn.cdc.gov/Nchs/Nhanes/1999-2000/TELO_A.html).

### Mortality

All-cause and cardiovascular mortality data were abstracted from the 1999–2002 NHANES public-use linked mortality files (LMF), which captured the vital status and cause of death of survey participants from survey participation (1999–2002) up to 31 December 2015. We examined all-cause mortality, as well as mortality due to cerebrovascular and cardiovascular diseases. Cardiovascular and cerebrovascular mortality was defined by International Classification of Diseases, 10th Edition, Clinical Modification System codes I00–I78 derived from death-certificate data. Detailed mortality variables for participants can be found on the website (https://www.cdc.gov/nchs/data-linkage/mortality-public.htm).

### Statistical analysis

Participants were classified into three groups (low, middle, high) according to MLT. All the continuous variables were presented as mean ± standard deviation, and categorical variables were presented in frequency or as a percentage. The One-Way Anova, Kruskal–Wallis Rank Sum Test, Fisher test and chi-square tests assessed subgroup differences. We used multivariate Cox proportional hazards regression to estimate the risk of all-cause, cerebrovascular and cardiovascular mortality and hazard ratios (HRs) and 95% confidence intervals (CIs) were shown. Censoring was done at the date of death or 31 December 2015, whichever came first. Model I only included MLT. The model II was adjusted for age, gender and BMI. The model III was further adjusted for SBP, CRP, TC, HDL-C, alcohol, smoking, marriage, poverty income ratio, education, race, diabetes, hypertension, cardiovascular diseases, eGFR, the use of antihypertensive drugs, lipid lowering drugs and anti-diabetic drugs. Kaplan–Meier survival curves for incidence of all-cause mortality between difference blood pressure groups were plotted and compared by the log-rank test. Subgroup analyses were conducted by stratifying age (<65 and ≥65 years), gender (female and male), BMI (<25 and ≥25 kg/m^2^), eGFR (<90 and ≥90 ml/min/1.73 m^2^), history of diabetes (yes and no), hypertension (yes and no) or cardiovascular disease (yes and no) at baseline. In addition, we used generalized additive model (GAM) to identify the non-linear relationship and the likelihood ration test was performed. If the non-linear correlation was observed, a two-piecewise linear regression model was performed to calculate the threshold effect of the MLT on mortality in terms of the smoothing plot. A two-sided *P*<0.05 was considered statistically significant. All statistical analyses were performed using R version 3.3.2 (R Foundation for Statistical Computing, Vienna, Austria).

## Results

### Baseline characteristics

There were total 7827 subjects in the study and 3771 (48.18%) were males. The characteristics of all participants are shown in Table 1. Briefly, median values of MLT in the lowest to highest quartiles were 0.77 ± 0.09, 1.00 ± 0.06 and 1.32 ± 0.26 T/S ratio. During the mean follow-up period of 158.26 ± 44.71 months, there were 1876 (23.97%), 87 (1.11%) and 243 (3.10%) onset of all-cause, cerebrovascular and cardiovascular mortality, respectively. In addition, there were significant subgroup differences in age, gender, BMI, SBP, FBG, CRP, TC, TG, LDL-C, HDL-C, alcohol, smoking status, marriage, poverty income ratio, education level, race, diabetes, hypertension, cardiovascular diseases, eGFR, the use of antihypertensive drugs, lipid lowering drugs and anti-diabetic drugs (all *P*<0.05).

**Table 1:** Baseline characteristics of participants

	Low (*n*=2609)	Middle (*n*=2609)	High (*n*=2609)	*P*
Age (years)				<0.001
<45	595 (22.81%)	1211 (46.42%)	1723 (66.04%)	
≥45, <65	851 (32.62%)	820 (31.43%)	619 (23.73%)	
≥65	1163 (44.58%)	578 (22.15%)	267 (10.23%)	
BMI (kg/m^2^)	28.61 ± 6.07	28.41 ± 6.11	27.97 ± 6.26	<0.001
SBP (mmHg)	122.25 ± 15.77	118.91 ± 15.41	116.91 ± 14.45	<0.001
DBP (mmHg)	70.36 ± 14.74	70.74 ± 12.74	70.28 ± 12.61	0.498
FBG (mmol/l)	109.57 ± 37.88	104.54 ± 38.08	98.93 ± 29.16	<0.001
TC (mg/dl)	207.59 ± 42.32	205.22 ± 41.67	200.10 ± 42.50	<0.001
TG (mg/dl)	159.99± 105.70	156.08 ± 157.46	138.90 ± 108.10	<0.001
LDLC (mg/dl)	124.53 ± 33.37	124.79 ± 35.49	121.25 ± 36.33	0.029
HDLC (mg/dl)	50.95 ± 15.62	51.66 ± 15.84	52.25 ± 15.46	0.011
eGFR (ml/min/1.73 m^2^)	90.19 ± 36.78	97.55 ± 35.98	106.11 ± 45.58	<0.001
CRP (mg/dl)	0.78 ± 1.60	0.64 ± 1.33	0.65 ± 1.35	<0.001
Alcohol (g)	8.10 ± 32.36	9.96 ± 32.58	11.13 ± 34.72	0.005
MTL (T/S ratio)	0.77 ± 0.09	1.00 ± 0.06	1.32 ± 0.26	<0.001
Gender (*n*, %)				<0.001
Male	1397 (53.55%)	1203 (46.11%)	1171 (44.88%)	
Female	1212 (46.45%)	1406 (53.89%)	1438 (55.12%)	
Smoking (*n*, %)				<0.001
Non-smoker	1238 (47.51%)	1337 (51.36%)	1441 (55.36%)	
Ex-smoker	878 (33.69%)	705 (27.08%)	518 (19.90%)	
Current smoker	490 (18.80%)	561 (21.55%)	644 (24.74%)	
Marriage (*n*, %)				<0.001
Married	1572 (61.94%)	1478 (59.48%)	1303 (53.58%)	
Single	697 (27.46%)	510 (20.52%)	359 (14.76%)	
Never married	176 (6.93%)	348 (14.00%)	600 (24.67%)	
Others	93 (3.66%)	149 (6.00%)	170 (6.99%)	
Educational level (*n*, %)				<0.001
Less than high school	1041 (39.99%)	841 (32.28%)	758 (29.08%)	
High school diploma	586 (22.51%)	597 (22.92%)	630 (24.17%)	
More than high school	976 (37.50%)	1167 (44.80%)	1219 (46.76%)	
Race/ethnicity (*n*, %)				<0.001
Black	356 (13.65%)	406 (15.56%)	571 (21.89%)	
Mexican American	637 (24.42%)	674 (25.83%)	565 (21.66%)	
Other Hispanic	126 (4.83%)	122 (4.68%)	169 (6.48%)	
Other race/ethnicity	1490 (57.11%)	1407 (53.93%)	1304 (49.98%)	
Diabetes (*n*, %)				<0.001
No	2169 (84.23%)	2318 (89.57%)	2398 (92.44%)	
Yes	406 (15.77%)	270 (10.43%)	196 (7.56%)	
Hypertension (*n*, %)				<0.001
No	1585 (60.94%)	1808 (69.75%)	1994 (76.90%)	
Yes	1016 (39.06%)	784 (30.25%)	599 (23.10%)	
Cardiovascular diseases (*n*, %)				<0.001
No	2410 (93.37%)	2509 (96.61%)	2533 (97.35%)	
Yes	171 (6.63%)	88 (3.39%)	69 (2.65%)	
Antihypertensive drugs (*n*, %)				<0.001
No	1846 (70.76%)	2128 (81.56%)	2277 (87.27%)	
Yes	763 (29.24%)	481 (18.44%)	332 (12.73%)	
Lipid lowering drugs (*n*, %)				<0.001
No	2332 (89.38%)	2445 (93.71%)	2499 (95.78%)	
Yes	277 (10.62%)	164 (6.29%)	110 (4.22%)	
Anti-diabetic drugs (*n*, %)				<0.001
No	2388 (91.53%)	2462 (94.37%)	2503 (95.94%)	
Yes	221 (8.47%)	147 (5.63%)	106 (4.06%)	
All-cause mortality (*n*, %)				<0.001
No	1592 (61.02%)	2063 (79.16%)	2292 (87.88%)	
Yes	1017 (38.98%)	543 (20.84%)	316 (12.12%)	
Cardiovascular mortality (*n*, %)				<0.001
No	2465 (94.48%)	2545 (97.55%)	2574 (98.66%)	
Yes	144 (5.52%)	64 (2.45%)	35 (1.34%)	
Cerebrovascular mortality				<0.001
No	2551 (97.78%)	2588 (99.20%)	2601 (99.69%)	
Yes	58 (2.22%)	21 (0.80%)	8 (0.31%)	

Data are expressed as mean ± SD of three groups. Abbreviation: Q, quartile.

### The relationship between MTL and all-cause, cerebrovascular and cardiovascular mortality

**Figure 2:**
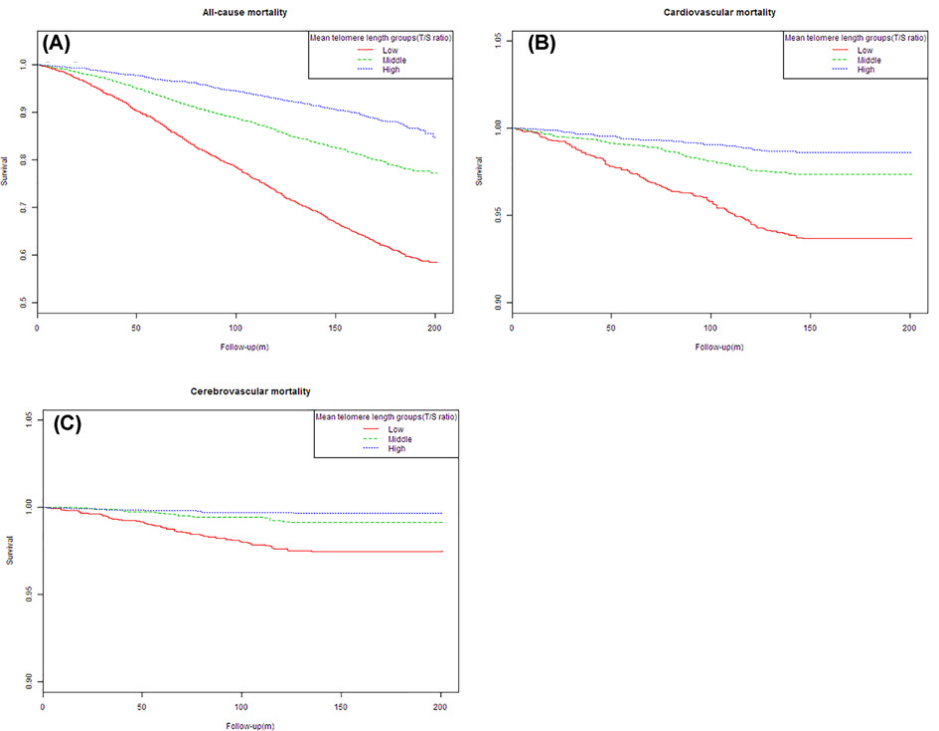
Kaplan–Meier estimated cumulative survival curves based on groups of MTL

As shown in Table 2, when treating MLT as a continuous variable, MLT was inversely associated with all-cause (HR = 0.08, 95% CI: 0.06, 0.10; *P*<0.0001), cerebrovascular (HR = 0.01, 95% CI: 0.00, 0.04; *P*<0.0001) and cardiovascular (HR = 0.05, 95% CI: 0.03, 0.09; *P*<0.0001) mortality in Model I. However, in the fully adjusted model (Model III), MLT showed no significant association with all-cause 0.78 (95% CI: 0.55, 1.10; *P*=0.1534), cerebrovascular 1.21 (95% CI: 0.16, 9.24; *P*=0.8547) and cardiovascular mortality 0.45 (95% CI: 0.14, 1.41; *P*=0.1707). When treating MLT as a categorical variable (MLT-low group as reference), in the fully adjusted model, high level of MLT did not associate with the risk of all-cause 0.86 (95% CI: 0.70, 1.05; *P*=0.1332) (*P*=0.127 for trend), cerebrovascular 0.75 (95% CI: 0.20, 2.81; *P*=0.6729) (*P*=0.593 for trend) and cardiovascular mortality 0.69 (95% CI: 0.33, 1.42; *P*=0.3077) (*P*=0.421 for trend). However, Kaplan–Meier survival curves demonstrated that there were significant differences in the prevalence rate of all-cause, cerebrovascular and cardiovascular mortality among MLT groups (Figure 2).

**Table 2:** Multivariate Cox regression analysis of MTL with all-cause, cardiovascular and cerebrovascular mortality

All-cause mortality	Model I	Model II	Model III
	HR (95% CI) *P*	HR (95% CI) *P*	HR (95% CI) *P*
MTL (T/S ratio)	0.08 (0.06, 0.10) <0.0001	0.75 (0.60, 0.94) 0.0120	0.78 (0.55, 1.10) 0.1534
MTL groups (T/S ratio)	HR (95% CI) *P*	HR (95% CI) *P*	HR (95% CI) *P*
Low	1.0	1.0	1.0
Middle	0.48 (0.43, 0.53) <0.0001	0.90 (0.81, 1.01) 0.0755	0.87 (0.74, 1.03) 0.1132
High	0.26 (0.23, 0.30) <0.0001	0.85 (0.74, 0.97) 0.0203	0.86 (0.70, 1.05) 0.1332
*P* for trend	<0.001	0.012	0.127
Cardiovascular mortality	HR (95% CI) *P*	HR (95% CI) *P*	HR (95% CI) *P*
MTL (T/S ratio)	0.05 (0.03, 0.09) <0.0001	0.71 (0.37, 1.37) 0.3095	0.45 (0.14, 1.41) 0.1707
MTL groups (T/S ratio)	HR (95% CI) *P*	HR (95% CI) *P*	HR (95% CI) *P*
Low	1.0	1.0	1.0
Middle	0.41 (0.30, 0.55) <0.0001	0.85 (0.62, 1.17) 0.3128	1.01 (0.61, 1.66) 0.9799
High	0.21 (0.15, 0.31) <0.0001	0.82 (0.54, 1.25) 0.3573	0.69 (0.33, 1.42) 0.3077
*P* for trend	<0.001	0.360	0.421
Cerebrovascular mortality	HR (95% CI) P	HR (95% CI) P	HR (95% CI) P
MTL (T/S ratio)	0.01 (0.00, 0.04) <0.0001	0.25 (0.07, 0.87) 0.0294	1.21 (0.16, 9.24) 0.8547
MTL groups (T/S ratio)	HR (95% CI) P	HR (95% CI) P	HR (95% CI) P
Low	1.0	1.0	1.0
Middle	0.34 (0.20, 0.55) <0.0001	0.87 (0.51, 1.48) 0.6009	0.75 (0.30, 1.89) 0.5444
High	0.12 (0.06, 0.26) <0.0001	0.44 (0.17, 1.14) 0.0912	0.75 (0.20, 2.81) 0.6729
*P* for trend	<0.001	0.130	0.593

Data are expressed as HR and 95% CI.

Model I adjusted for None.

Model II adjusted for age, gender and BMI.

Model III adjusted for age, gender, BMI, SBP, CRP, TC, HDL-C, alcohol, smoking, marriage, poverty income ratio, education, race, diabetes, hypertension, cardiovascular diseases, eGFR, antihypertensive drugs, lipid lowering drugs and anti-diabetic drugs.

### Subgroup analysis of MTL with all-cause, cardiovascular and cerebrovascular mortality

Table 3 demonstrates the multivariate Cox regression analysis association between MLT with all-cause, cardiovascular and cerebrovascular mortality in subgroups. When treating MLT as a continuous variable, we only found that MLT was inversely associated with all-cause mortality among males (HR = 0.53, 95% CI: 0.30, 0.94; *P*=0.0302), participants with eGFR < 90 ml/min/1.73 m^2^ (HR = 0.54, 95% CI: 0.30, 0.98; *P*=0.0417) or age < 65 years (HR = 0.45, 95% CI: 0.28, 0.75; *P*=0.0021) or age ≥ 65 years (HR = 0.27, 95% CI: 0.12, 0.58; *P*=0.0009). For cardiovascular mortality, the association was significant among those aged < 65 years (HR = 0.04, 95% CI: 0.01, 0.29; *P*=0.0015).

**Table 3:** Subgroup analysis of MTL with all-cause, cardiovascular and cerebrovascular mortality

	Number	All-cause mortality	Cardiovascular mortality	Cerebrovascular mortality
		HR (95% CI) *P*	HR (95% CI) *P*	HR (95% CI) *P*
Gender				
Male	3771	0.53 (0.30, 0.94) 0.0302	0.49 (0.11, 2.13) 0.3421	0.34 (0.02, 5.44)
0.4465				
Female	4056	1.23 (0.67, 2.27) 0.4992	0.40 (0.06, 2.64) 0.3408	6.84 (0.53, 88.52) 0.1410
*P* interaction		0.0384	0.8622	0.1130
Diabetes				
No	6885	0.82 (0.51, 1.31) 0.4004	0.52 (0.14, 1.92) 0.3274	0.89 (0.10, 8.15) 0.9215
Yes	872	0.56 (0.18, 1.68) 0.2995	0.24 (0.01, 4.05) 0.3253	17.26 (0.18, 1629.91) 0.2195
*P* interaction		0.5232	0.6247	0.2718
Hypertension				
No	5387	0.76 (0.44, 1.31) 0.3229	0.65 (0.10, 4.22) 0.6472	0.67 (0.03, 13.28) 0.7951
Yes	2399	0.79 (0.40, 1.57) 0.5071	0.37 (0.09, 1.62) 0.1876	2.50 (0.18, 33.86) 0.4907
*P* interaction		0.9215	0.6427	0.5011
CVD				
No	7452	0.74 (0.47, 1.16) 0.1835	0.43 (0.12, 1.54) 0.1932	1.61 (0.21, 12.48) 0.6493
Yes	328	1.68 (0.30, 9.41) 0.5572	0.68 (0.03, 15.27) 0.8062	0.00 (0.00, 14373.05) 0.4724
		0.3647	0.7883	0.3915
Age				
<65	5819	0.45 (0.28, 0.75) 0.0021	0.04 (0.01, 0.29) 0.0015	2.22 (0.20, 24.15) 0.5123
≥65	2008	0.27 (0.12, 0.58) 0.0009	0.79 (0.18, 3.45) 0.7552	0.06 (0.00, 1.30) 0.0731
*P* interaction		0.2544	0.0160	0.0747
BMI				
<25	2402	0.69 (0.33, 1.43) 0.3179	0.48 (0.07, 3.10) 0.4420	3.04 (0.10, 91.94) 0.5226
≥25	5176	0.83 (0.50, 1.39) 0.4765	0.44 (0.10, 1.93) 0.2749	1.01 (0.09, 11.83) 0.9968
*P* interaction		0.6664	0.9369	0.5979
eGFR				
<90	3683	0.54 (0.30, 0.98) 0.0417	0.47 (0.12, 1.91) 0.2932	0.36 (0.02, 6.50) 0.4884
≥90	4135	1.13 (0.63, 2.05) 0.6789	0.39 (0.05, 3.11) 0.3773	5.34 (0.43, 65.87) 0.1911
P interaction		0.0685	0.8816	0.1583

Data are expressed as HR and 95% CI.

Adjusted for age, gender, BMI, SBP, CRP, TC, HDL-C, alcohol, smoking, marriage, poverty income ratio, education, race, diabetes, hypertension, cardiovascular diseases, eGFR, antihypertensive drugs, lipid lowering drugs and anti-diabetic drugs.

### The analyses of non-linear relationship

**Figure 3:**
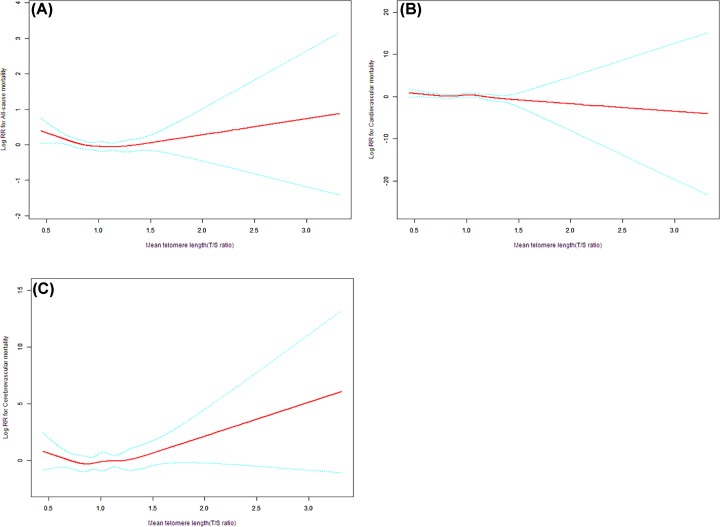
The association of MTL with all-cause, cerebrovascular and cardiovascular mortality Data are expressed as mean (95% CI). The red line represents RR and the green line represents 95% CI. Abbreviation: RR, relative risk.

As shown in Table 4, two-piecewise linear regression model suggested that the inflection point of all-cause, cerebrovascular and cardiovascular mortality were 0.94 T/S ratio, 0.92 T/S ratio and 1.22 T/S ratio, respectively. On the left and right of the inflection points, the HRs of all-cause mortality were 0.41 (95% CI: 0.21, 0.79; *P*=0.0083) and 1.25 (95% CI: 0.74, 2.13; *P*=0.4069), respectively. However, on both sides of the threshold, the HRs for cerebrovascular mortality were 0.08 (95% CI: 0.00, 2.36; *P*=0.1412) and 9.90 (95% CI: 0.75, 130.17; *P*=0.0812), while that for cardiovascular mortality were 0.61 (95% CI: 0.16, 2.30; *P*=0.4675) and 0.01 (95% CI: 0.00, 83.57; *P*=0.3187), respectively. As shown in Figure 3, GAM revealed that the relationship between MLT and all-cause mortality was non-linear but had no significant relationship with cerebrovascular and cardiovascular mortality after adjustment for potential variables.

**Table 4:** The results of two-piecewise linear regression model between MTL and all-cause, cardiovascular and cerebrovascular mortality

Outcome	All-cause mortality	Cardiovascular mortality	Cerebrovascular mortality
	HR (95% CI) *P*	HR (95% CI) *P*	HR (95% CI) *P*
Cutoff value (T/S ratio)	0.94	1.22	0.92
<Cut-off value	0.41 (0.21, 0.79) 0.0083	0.61 (0.16, 2.30) 0.4675	0.08 (0.00, 2.36) 0.1412
≥Cut-off value	1.25 (0.74, 2.13) 0.4069	0.01 (0.00, 83.57) 0.3187	9.90 (0.75, 130.17) 0.0812
*P* for log likelihood ratio test	0.029	0.326	0.076

Data are expressed as HR and 95% CI.

Effect: all-cause, cardiovascular and cerebrovascular mortality, Cause: MTL. Adjusted for age, gender, BMI, SBP, CRP, TC, HDL-C, alcohol, smoking, marriage, poverty income ratio, education, race, diabetes, hypertension, cardiovascular diseases, eGFR, antihypertensive drugs, lipid lowering drugs and anti-diabetic drugs.

## Discussion

In the present study, our main findings were as follows: (1) MLT was non-linearly related to all-cause mortality in a large, nationally representative sample of U.S. adults; (2) MLT has no statistically significant relationship with cardiovascular and cerebrovascular mortality; (3) when MLT was less than 0.94 T/S ratio, it could increase the risk of all-cause mortality by 41% for every 0.1 T/S ratio shortening of telomere. However, when it was higher than 0.94 T/S ratio, there was no statistically significant relationship between MLT and all-cause mortality.

In the present study, there was no statistically significant relationship between all-cause mortality when MLT was a categorical variable. A study from Swedish elderly men indicated that MTL attrition did not predict all-cause mortality [19]. Furthermore, Kotsopoulos et al. [20] provided preliminary evidence that telomere length likely did not predict mortality after a diagnosis of ovarian cancer. In addition, among a community-based cohort of African Americans, after a median follow-up of 9 years, longer telomere length was associated with a lower risk of incident ischemic stroke and total mortality in age- and sex-adjusted models, but these associations were no longer significant in fully adjusted models [21]. However, there were also a large number of studies that suggested telomere length relating to all-cause mortality in different population [22,23].

According to the present study, we also revealed that the high MLT group could have 31% reduction in the risk of cardiovascular mortality and 25% less risk of cerebrovascular mortality compared with the low MLT group, but these associations were no longer significant after adjustment for potential variables. These findings were in agreement with previous research that there was no significant association between telomere length and cardiovascular or cerebrovascular mortality [24,25]. In contrast, a previous study found a 60% decreased risk for cardiovascular mortality in those who shortened their telomeres [19]. Burnett-Hartman et al. [26] found that telomere biology and associated genes may play a role in cardiovascular disease-related death, particularly among women.

The possible reasons for the different results of our research and previous research may due to differential ethnicity background and covariates being adjusted. Most of the previous studies only adjusted for anthropometric and demographic features, but we adjusted more mortality-related additional confounding variables such as eGFR, CRP, blood lipids, FBG and taking drugs. We speculated that some of the confounding factors associated with mortality may have a certain impact on the results. Furthermore, telomere variation, telomere measurement techniques and age at measurement contributed to the heterogeneity and may also have an effect on the conclusion of some studies. Our results highlight the need to confirm these findings and further explore the reasons behind the heterogeneous observation.

Several potential limitations should be taken into consideration. First, the analysis method of the qPCR was full of high variability, so there may be errors in the measurement of telomere length. Second, the study population was solely from United States, so the conclusions of this research may not be extrapolated to other populations. Third, both all-cause, cerebrovascular and cardiovascular mortality are multifactor-related end points, although we have adjusted multiple potential confounders. The present study has not adjusted for some other death-related confounders such as tumor history, weakness, mental condition, exercise and sleep.

Despite these limitations, the present study has several strengths. First, the sample size of the present study was comparatively large from a well-designed population-based cohort study. Second, the research results have sufficient statistical effects due to the long follow-up period. Third, numerous confounding factors associated with mortality, including socio-demographic factors, lipids parameters, blood pressure, BMI, FBG, CRP, previous history of diseases, taking drugs, alcohol and smoking status were adjusted in the present study.

In the present study we show the non-linear association between MLT and all-cause mortality. MLT inversely associated with all-cause mortality when MLT was less than 0.94 T/S ratio. However, there is no significant association with MLT cerebrovascular and cardiovascular mortality.

## Abbreviations

BMI: body mass index
CDC: Centers for Disease Control and Prevention
CI: confidence interval
CRP: C-reactive protein
eGFR: estimated glomerular filtration rate
FBG: fasting blood glucose
GAM: generalized additive model
HDL-C: high-density lipoprotein cholesterol
HR: hazard ratio
LDL-C: low-density lipoprotein cholesterol
MLT: mean telomere length
NHANES: National Health and Nutrition Examination Survey
qPCR: quantitative polymerase chain reaction
SBP: systolic blood pressure
SCr: serum creatinine
TC: total cholesterol
TG: triglyceride

## References

[1] Wang Q., Zhan Y., Pedersen N.L., Fang F.and Hagg S. (2018) Telomere length and all-cause mortality: a meta-analysis. Ageing Res. Rev. 48, 11–20 10.1016/j.arr.2018.09.002 30254001

[2] Blackburn E.H. (2000) Telomere states and cell fates. Nature 408, 53–6 10.1038/35040500 11081503

[3] Blackburn E.H., Epel E.S.and Lin J. (2015) Human telomere biology: a contributory and interactive factor in aging, disease risks, and protection. Science 350, 1193–8 10.1126/science.aab3389 26785477

[4] Toupance S., Labat C., Temmar M., Rossignol P., Kimura M., Aviv A. et al. (2017) Short telomeres, but not telomere attrition rates, are associated with carotid atherosclerosis. Hypertension 70, 420–425 10.1161/HYPERTENSIONAHA.117.09354 28630210 PMC5903283

[5] Jenkins E.C., Marchi E.J., Velinov M.T., Ye L., Krinsky-McHale S.J., Zigman W.B. et al. (2017) Longitudinal telomere shortening and early Alzheimer’s disease progression in adults with down syndrome. Am. J. Med. Genet. B Neuropsychiatr. Genet. 174, 772–778 10.1002/ajmg.b.32575 28856789

[6] Ding H., Chen C., Shaffer J.R., Liu L., Xu Y., Wang X. et al. (2012) Telomere length and risk of stroke in Chinese. Stroke 43, 658–63 10.1161/STROKEAHA.111.637207 22343648

[7] Brouilette S.W., Moore J.S., McMahon A.D., Thompson J.R., Ford I., Shepherd J. et al. (2007) Telomere length, risk of coronary heart disease, and statin treatment in the West of Scotland Primary Prevention Study: a nested case-control study. Lancet 369, 107–14 10.1016/S0140-6736(07)60071-3 17223473

[8] Loprinzi P.D. (2015) Cardiorespiratory capacity and leukocyte telomere length among adults in the United States. Am. J. Epidemiol. 182, 198–201 10.1093/aje/kwv056 26153476

[9] Xu X., Qu K., Pang Q., Wang Z., Zhou Y.and Liu C. (2016) Association between telomere length and survival in cancer patients: a meta-analysis and review of literature. Front. Med. 10, 191–203 10.1007/s11684-016-0450-2 27185042

[10] Gao X., Zhang Y., Mons U.and Brenner H. (2018) Leukocyte telomere length and epigenetic-based mortality risk score: associations with all-cause mortality among older adults. Epigenetics 13, 846–857 10.1080/15592294.2018.1514853 30152726 PMC6224222

[11] Marioni R.E., Harris S.E., Shah S., McRae A.F., von Zglinicki T., Martin-Ruiz C. et al. (2016) The epigenetic clock and telomere length are independently associated with chronological age and mortality. Int. J. Epidemiol. 45, 424–432 10.1093/ije/dyw041 27075770 PMC4864882

[12] Jin M., Lee E.C., Ra S.W., Fishbane N., Tam S., Criner G.J. et al. (2018) Relationship of absolute telomere length with quality of life, exacerbations, and mortality in COPD. Chest 154, 266–273 10.1016/j.chest.2018.05.022 30017346

[13] Goglin S.E., Farzaneh-Far R., Epel E.S., Lin J., Blackburn E.H.and Whooley M.A. (2016) Change in leukocyte telomere length predicts mortality in patients with stable coronary heart disease from the Heart and Soul Study. PLoS ONE 11, e0160748 10.1371/journal.pone.0160748 27783614 PMC5081189

[14] Bonfigli A.R., Spazzafumo L., Prattichizzo F., Bonafe M., Mensa E., Micolucci L. et al. (2016) Leukocyte telomere length and mortality risk in patients with type 2 diabetes. Oncotarget 7, 50835–50844 10.18632/oncotarget.10615 27437767 PMC5239440

[15] Loprinzi P.D.and Loenneke J.P. (2018) Leukocyte telomere length and mortality among U.S. adults: effect modification by physical activity behaviour. J. Sports Sci. 36, 213–219 10.1080/02640414.2017.1293280 28282748

[16] Doherty J.A., Grieshober L., Houck J.R., Barnett M.J., Tapsoba J.D., Thornquist M. et al. (2018) Telomere length and lung cancer mortality among heavy smokers. Cancer Epidemiol. Biomarkers Prev. 27, 829–837 10.1158/1055-9965.EPI-17-1183 29743162 PMC6035074

[17] Stevens L.A., Coresh J., Greene T.and Levey A.S. (2006) Assessing kidney function–measured and estimated glomerular filtration rate. N. Engl. J. Med. 354, 2473–83 10.1056/NEJMra054415 16760447

[18] Scinicariello F., Feroe A.G.and Attanasio R. (2016) Urinary phthalates and leukocyte telomere length: an analysis of NHANES 1999-2002. Ebiomedicine 6, 96–102 10.1016/j.ebiom.2016.02.027 27211552 PMC4856743

[19] Yuan X., Kronstrom M., Hellenius M.L., Cederholm T., Xu D.and Sjogren P. (2018) Longitudinal changes in leukocyte telomere length and mortality in elderly Swedish men. Aging 10, 3005–3016 10.18632/aging.101611 30375983 PMC6224259

[20] Kotsopoulos J., Prescott J., De Vivo I., Fan I., Mclaughlin J., Rosen B. et al. (2014) Telomere length and mortality following a diagnosis of ovarian cancer. Cancer Epidemiol. Biomarkers Prev. 23, 2603–6 10.1158/1055-9965.EPI-14-0885 25159293 PMC4221534

[21] Mwasongwe S., Gao Y., Griswold M., Wilson J.G., Aviv A., Reiner A.P. et al. (2017) Leukocyte telomere length and cardiovascular disease in African Americans: The Jackson Heart Study. Atherosclerosis 266, 41–47 10.1016/j.atherosclerosis.2017.09.016 28950166 PMC5671898

[22] Stefler D., Malyutina S., Maximov V., Orlov P., Ivanoschuk D., Nikitin Y. et al. (2018) Leukocyte telomere length and risk of coronary heart disease and stroke mortality: prospective evidence from a Russian cohort. Sci. Rep. 8, 16627 10.1038/s41598-018-35122-y 30413768 PMC6226519

[23] Dean S.G., Zhang C., Gao J., Roy S., Shinkle J., Sabarinathan M. et al. (2017) The association between telomere length and mortality in Bangladesh. Aging 9, 1537–1551 10.18632/aging.101246 28630379 PMC5509454

[24] Svensson J., Karlsson M.K., Ljunggren O., Tivesten A., Mellstrom D.and Moverare-Skrtic S. (2014) Leukocyte telomere length is not associated with mortality in older men. Exp. Gerontol. 57, 6–12 10.1016/j.exger.2014.04.013 24793325

[25] Rode L., Nordestgaard B.G.and Bojesen S.E. (2015) Peripheral blood leukocyte telomere length and mortality among 64,637 individuals from the general population. J. Natl. Cancer Inst. 107, djv074 10.1093/jnci/djv074 25862531

[26] Burnett-Hartman A.N., Fitzpatrick A.L., Kronmal R.A., Psaty B.M., Jenny N.S., Bis J.C. et al. (2012) Telomere-associated polymorphisms correlate with cardiovascular disease mortality in Caucasian women: the Cardiovascular Health Study. Mech. Ageing Dev. 133, 275–81 10.1016/j.mad.2012.03.002 22449406 PMC3391009

